# ABO Blood Grouping Mismatch in Hematopoietic Stem Cell Transplantation and Clinical Guides

**Published:** 2018-10-01

**Authors:** Negin Shokrgozar, Gholamhossein Tamaddon

**Affiliations:** 1 School of Paramedical Sciences, Shiraz University of Medical Sciences, Shiraz, Iran; 2 Diagnostic Laboratory Sciences and Technology Research Center, School of Paramedical Sciences, Shiraz University of Medical Sciences, Shiraz, Iran

**Keywords:** Hematopoietic stem cell transplantation, Major ABO mismatch, Minor ABO mismatch, Transfusion strategy

## Abstract

Hematopoietic stem cell transplantation (HSCT) is a useful treatment. In contrast to solid organ transplantations, the use of ABO blood group mismatch is acceptable in HSCT. Immediate or late hemolytic reactions, pure red cell aplasia, delayed red blood cell recovery, and graft-versus -host disease are the results of this situation. This review shows the consequences of ABO-mismatched HSCT and its impacts on HSCT parameters, as well as providing clinical guides in this situation.

## Introduction

 Hematopoietic stem cell transplantation (HSCT) is the graft of hematopoietic stem cells that can be extracted from bone marrow, peripheral blood or umbilical cord blood. This transplantation can provide the cure for malignant and non-malignant diseases such as leukemia, solid tumors, aplastic anemia, and thalassemia^1^^,^^2^.

In contrast to solid organ transplantations, HSCT can be performed across ABO incompatibility^3^_._ ABO groups are inherited independently from human leukocyte antigens (HLA), hence, ABO incompatibility between donor and recipient is observed in 30-40% of patients undergoing HSCT^4^. Human leukocyte antigens (HLA) and ABO blood group antigens are coded by genes on chromosomes 6 and 9^5^. ABO blood group antigens include A, B, and O. These antigens are on RBCs and each person has antibodies in serum or plasma against antigens that do not exist on RBCs. For example people with O blood group have anti-A, anti-B, and anti-AB in their serum or plasma^6^ (Table1).

Three types of ABO incompatibility have been identified as major, minor and bidirectional. Major ABO incompatibility occurs by antidonor isoagglutinins; for instance, when the recipient has O-blood group and the donor has A, B, or AB-blood group. In minor ABO incompatibility, donor B lymphocytes produce antirecipient isoagglutinins; for example, when the donor has O-blood group and recipient has A, B, or AB-blood group. Bidirectional ABO incompatibility occurs when the donor and recipient have isohemagglutinins (IHAs) against each other^7^.

Before transplantation, we can decrease antibody titers by plasma or whole blood exchange^8^. HSCT with major ABO incompatibility can be more complicated compared to peripheral blood stem cell (PBSC) since grafts from bone marrow contain high amount of red blood cells^9^.

**Table1:** ABO blood groups and serum antibodies

**Blood Groups**	**Cell Antigen**	**Serum Antibodies**
**A**	A	Anti-B
**B**	B	Anti-A
**AB**	AB	None
**O**	None	Anti-A,B,AB

**Definition and complications in ABO-mismatched HSCT**

**Major ABO-mismatched HSCT**

Major ABO-mismatched HSCT can cause hemolysis of donor’s erythrocytes by recipient’s IHAs^10^. In bone marrow derived grafts, hemolysis is more common than PBSC due to the high amount of erythrocytes in bone marrow^11^. In major ABO-mismatched HSCT, hemolysis can be prevented by removing erythrocytes from graft. Insignificant hemolysis can also occur during erythrocyte engraftment due to destruction of erythrocytes containing donor’s antigens by means of recipient’s IHAs^12^. Finally, these reactions cause pure red cell aplasia (PRCA) in the majority of patients who had major ABO-mismatched HSCT ^13^. Antibody titers can be diminished in major ABO-mismatched HSCT by plasma or whole blood exchange before engraftment^8^.

**Minor ABO-mismatched HSCT (passenger lymphocytes syndrome)**

**Fig.1:**
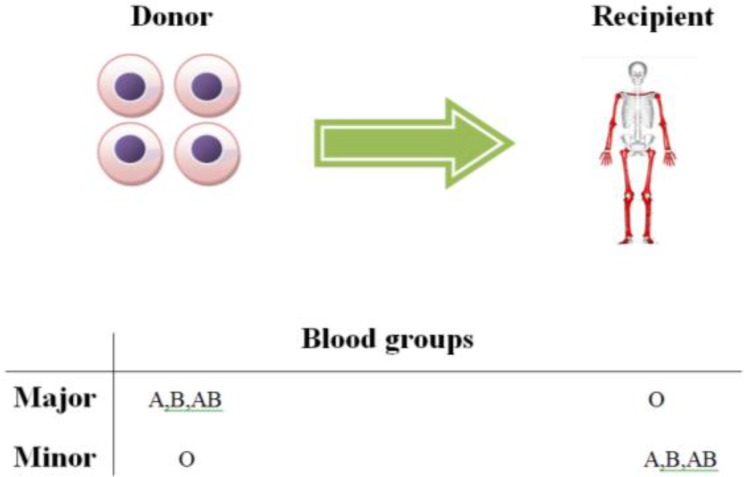
ABO incompatibility: Isohemagglutinins in recipient or donor can cause major or minor ABO incompatibility in HSCT.

About 7-14 days after the infusion of graft, hemolysis occurs due to donor’s IHAs against recipient’s erythrocytes^14^. This immediate hemolysis can be more severe than major ABO-mismatched HSCT that usually decreases after 5-10 days. In this situation, direct antiglobulin test (DAT) is usually positive against recipient’s erythrocytes antigens. A second hemolytic reaction occurs due to immunization of donor’s B lymphocytes, which is called passenger lymphocytes (PL) and production of IHAs against recipient’s erythrocytes, which is called “delayed hemolysis”. An important factor in development of PL syndrome is PBSC-derived grafts due to high lymphocyte content^15^. In minor ABO-mismatched HSCT, IHAs can be removed from the graft by various techniques. There is a significant association between minor ABO-mismatched HSCT and increased risk of acute graft-versus-host disease (aGVHD) in patients^16^ (Figure1).

**Effects of ABO-mismatch on HSCT parameters**

These parameters include engraftment, GVHD and relapse^17^.

**Engraftment**

A comparison between ABO-identical groups and each mismatched group, only major ABO incompatibility indicated a delay in blood cells recovery such as red blood cells, neutrophils and platelets^18^. However, the effect of ABO-mismatched HSCT on erythrocyte engraftment has been shown more clearly in several studies. A multinational study in 232 centers by CIBMTR (Center for International Blood and Marrow Research) compared a large number of patients who had ABO-mismatched HSCT (N=995) with ABO-matched HSCT (N=2108). The study groups included children and adults (age range: 1–69 years for patients, and 1–68 years for donors). Almost all of the patients received transplantation from their related donors after myeloablative conditioning. This study revealed that having major-ABO-mismatched donor can result in prolonged erythrocyte transfusion requirement and a longer time to neutrophil engraftment^4^.

Delayed erythrocyte engraftment is observed only in patients with bone marrow-derived grafts, which is due to the use of immunosuppressive drugs such as mycophenolate mofetil that suppresses antibody production by B lymphocytes^19^.

**Pure red cell aplasia (PRCA)**

PRCA is a common complication after major ABO-mismatched HSCT that occurs in the absence of erythroid engraftment^20^. This situation commonly occurs between donor with A-blood group and recipient with O-blood group due to the presence of anti-donor IHAs, especially anti-A isohemagglutinin, against donor’s erythrocytes^21^. Before transplantation, IHAs can be reduced by plasma exchange or immunoadsorption that reduces the risk of PRCA^22^.

If anti-donor isohemagglutinins remain more than 60 days after HSCT, they can be removed by various techniques such as plasma exchange, immunoadsorption, immunosuppressive drugs, administration of donor leukocyte infusions (DLI), anti-lymphocyte, anti-thymocyte globulins and erythropoietin injection^13^^, ^^23^^-^^28^. In some cases, rituximab, a monoclonal antibody against CD20 positive B lymphocytes, is used ^29^. Bortezomib is a proteasome inhibitor that is usually used to treat plasma cell dyscrasias, and it can be used to treat PRCA after transplantation in patients with no response to different treatments such as immunosuppressive, corticosteroids and rituximab^30^.

**Transfusion strategy**

**Major ABO-mismatched HSCT**

A common complication associated with major ABO-mismatched HSCT is the transfusion of ABO incompatible erythrocytes that can cause hemolysis, acute renal failure and death^31^.

The transfusion strategy in ABO-mismatched cases should consider both the recipient and donor blood group systems^32^. If the titer of anti-donor IHAs is more than 32, especially in PBSC-derived grafts, erythrocyte volume should not be more than 20 ml in the graft^33^.

In major or bidirectional ABO-mismatched HSCT cases, transfusion of blood group O RBCs and blood group AB platelets is necessary. Transfusion of blood group O RBCs is necessary until anti-donor IHAs are undetectable in recipient’s blood sample. Since ABO antigens are also present on the platelet surface, consequently in group O patients with high amounts of anti-A IHAs, platelets of group A1 donors must be avoided^3^. Before transfusion, all the packed RBCs and platelets should be separated from plasma and irradiated at a dose of 30 Gy to prevent any risk of acute transfusion-induced GVHD^19^.

**Minor ABO-mismatched HSCT**

Graft plasma reduction can be useful to decrease the reaction between recipient’s erythrocytes and donor-derived IHAs. This method is useful when the titer of anti-A or anti-B is more than 1/256 in donor’s plasma^7^. In addition, erythrocytes can be exchanged using a cell separator. In this method, recipient’s erythrocytes can be separated through centrifugation and replaced by group O erythrocytes^34^.

After transplantation, it is necessary to transfuse patients with erythrocytes compatible with donor’s blood group^32^ (Table2).

**Table2:** Recommended transfusion support for recipients of ABO incompatible HSCT

	**Recipient**	**Donor**	**RBC and** **granulocyte** **components**	**Platelet and** **plasma** **components**
ABO major	O O O A B	A B AB AB AB	O O O A,O B,O	A,AB B,AB AB AB AB
ABO minor	A B AB AB AB	O O O A B	O O O A,O B,O	A,AB B,AB AB AB AB
ABO major and minor	A B	B A	O O	AB AB

**Clinical guides to ABO-incompatible HSCT**

**Major ABO incompatibility**

One strategy to prevent hemolysis is RBCs depletion from graft before transplantation. In this procedure, the overall progenitor cell content of the HPC product can be reduced, especially in cases that the source of stem cells is cord blood^35^^, ^^36^. Another method to prevent complications is reducing the recipient’s IHAs titer by various techniques such as plasma exchange or immunoadsorption columns^8^. The use of donor-type secretor plasma can be useful in reducing isohemagglutinins before transplantation. Although non-secretor plasma can also be used, the use of donor-type secretor plasma (i.e, donors who secrete A or B antigens into plasma and other body fluids) increases the chance of IHAs reduction after infusion. In this procedure, A or B antigens in secretor plasma bind to recipient’s anti-A/B isohemagglutinin which results in depletion of IHAs^37^^,^^38^. Also, donor-type RBCs can be used for this purpose, but there is a risk of hemolytic-type reactions in comparison to secretor plasma^22^. 

**Minor ABO incompatibility**

Plasma reduction cannot reduce B lymphocytes in marrow transplantation, hence, it has no effect on PLS. Rituximab is usually used to prevent GVHD and can reduce PLS occurrence^12^. PLS can also occur in solid organ transplantation if donor’s B lymphocytes are present in graft or when immunosuppression after transplantation does not have an antiproliferative agent^39^^,^^40^. Some studies suggest pretransplant red cell exchange to reduce incompatible donor RBCs before infusion, but this procedure is not widely acceptable^25^.

**Bidirectional ABO incompatibility**

In these cases, both major and minor incompatibility occurs, and complications of both types should be controlled. In this situation, patients require AB plasma products and group O RBCs^7^.

## Discussion

 The aim of this mini review was to indicate the consequences of ABO-mismatched HSCT and its impacts on HSCT parameters and to provide clinical guides in this situation. ABO incompatibility is not a barrier in hematopoietic stem cell transplantation^3^. According to previous studies, patients with major incompatibility are at high risk of immediate hemolysis due to anti donor IHAs, delayed engraftment and PRCA, which result in more RBC transfusion^41^. When donor has A blood group and recipient has O blood group, the incidence of PRCA is more common^20^. In patients with PRCA who do not respond to conventional treatments, infusions of human adipose tissue-derived mesenchymal stem cells (AMSC) can be a therapeutic option^42^. Kimura et al. observed poor overall survival in major incompatibility, and patients with major and minor incompatibility had higher risk of acute GVHD^18^. De Santis et al. reported that anti-A/B titer ≥32 of IgG class (but not IgM) is associated with higher RBC transfusion and antibody titer ≤16 could be a predictor of RBC transfusion requirement^43^. The most common preventive measures for major ABO incompatibility are depletion of RBCs from graft, reducing the titer of incompatible recipient’s IHAs, and the use of donor-type secretor plasma to reduce IHAs before transplantation^7^. It has been shown that 10-30 ml of incompatible RBCs can be tolerated by adults, and when it is less than 15 ml no significant hemolysis is seen^44^^,^^45^. Patients with minor ABO incompatibility are at high risk of massive immune hemolysis and aGVHD after HSCT^15^^, ^^16^_._ Grube et al. showed that ABO blood group mismatch has a significant impact on the outcome. Moreover, they found that minor-A and minor-AB ABO-mismatch represent a risk factor for increased transplant-related mortality after allo PBSCT^46^. Bolan in a study showed that hemolysis due to minor mismatched HSCT can be life-threatening and difficult to diagnose. They also suggested that the etiology of hemolysis is multifactorial such as the use of cyclosporin alone in the absence of an anti-proliferative agent for GVHD prophylaxis^15^. It seems that using rituximab and pretransplant red cell exchange are often helpful in managing minor ABO incompatibility^7^. After ABO-mismatched HSCT, transfusion strategies are so important and each institution must perform standard procedures to choose the ABO type of products transfused to recipients of ABO-incompatible transplants^47^. Before transplantation, blood components should be compatible with recipient, but after that, both recipient and donor must be considered ^33^. However, lack of knowledge and guidelines can endanger the recipients, which needs more investigation to prevent ABO incompatibility complications. 
